# Functional characterization of novel compound heterozygous missense *SLC5A5* gene variants causing congenital dyshormonogenic hypothyroidism

**DOI:** 10.3389/fendo.2024.1465176

**Published:** 2024-12-19

**Authors:** Gerardo Hernán Carro, Mariano Martín, Sofía Savy, Victoria Peyret, Romina Celeste Geysels, Francisco Andrés Montes, Carlos Eduardo Bernal Barquero, Valentina Ricci, María Eugenia Masnata, Ana María Masini-Repiso, Patricia Papendieck, Mariana Lorena Tellechea, Ana Elena Chiesa, Juan Pablo Nicola

**Affiliations:** ^1^ Departamento de Bioquímica Clínica, Facultad de Ciencias Químicas, Universidad Nacional de Córdoba, Córdoba, Argentina; ^2^ Centro de Investigaciones en Bioquímica Clínica e Inmunología, Consejo Nacional de Investigaciones Científicas y Técnicas (CIBICI-CONICET), Córdoba, Argentina; ^3^ División de Endocrinología, Hospital de Niños Dr. Ricardo Gutiérrez, Buenos Aires, Argentina; ^4^ Centro de Investigaciones Endocrinológicas Dr. César Bergadá, Consejo Nacional de Investigaciones Científicas y Técnicas (CEDIE-CONICET), Buenos Aires, Argentina

**Keywords:** congenital hypothyroidism, iodide transport defect, sodium iodide symporter (NIS), whole-exome sequencing, biallelic loss-of-function *SLC5A5* variants

## Abstract

**Introduction:**

The sodium/iodide symporter (NIS) mediates active iodide accumulation in the thyroid follicular cell. Biallelic loss-of-function variants in the NIS-coding *SLC5A5* gene cause congenital dyshormonogenic hypothyroidism due to a defect in the accumulation of iodide, which is required for thyroid hormonogenesis.

**Objective:**

We aimed to identify, and if so to functionally characterize, novel pathogenic *SLC5A5* gene variants in a patient diagnosed with severe congenital dyshormonogenic hypothyroidism characterized by undetectable radioiodide accumulation in a eutopic thyroid gland, as well as in the salivary glands.

**Methods:**

The coding region of the *SLC5A5* gene was sequenced using whole-exome sequencing. In silico analysis and in vitro functional characterization of missense *SLC5A5* gene variants were performed.

**Results:**

Proposita’s whole-exome sequencing revealed a novel pair of compound heterozygous missense variants in the *SLC5A5* gene, c.1,627G>A (p.G543R) and c.1,684T>A (p.L562M). The parents were heterozygous carriers of the variants as determined by Sanger sequencing of the *SLC5A5* gene. The p.G543R variant in the homozygous state has previously been associated with congenital hypothyroidism. The novel p.L562M variant was not reported in the Genome Aggregation Consortium dataset. In silico analysis of the pathogenic impact of the p.L562M variant yielded inconclusive results. Functional in vitro studies showed that the p.L562M variant reduces iodide accumulation due to defective expression of the mutant NIS protein at the plasma membrane. Notably, the aliphatic residue Leu at position 562 in the carboxy terminus of the protein, which is highly conserved in NIS orthologues, is required for NIS plasma membrane expression.

**Conclusions:**

We report novel compound heterozygous missense *SLC5A5* gene variants causing defective iodide accumulation, thus leading to congenital dyshormonogenic hypothyroidism.

## Introduction

Congenital hypothyroidism is the most common endocrine disorder in newborns, and among the most common preventable causes of intellectual disability. In Argentina, congenital hypothyroidism occurs with an incidence of 1:2,367 based on thyrotropin-based newborn screening programs (1). Genetic defects or environmental factors affecting thyroid hormonogenesis lead to congenital dyshormonogenic hypothyroidism (2). In particular, iodide transport defect is a rare autosomal recessive disorder caused by the inability of thyroid follicular cells to accumulate iodide, resulting in congenital dyshormonogenic hypothyroidism. An iodide transport defect is suspected when normal radioiodide accumulation in a eutopic thyroid gland, as well as, in the salivary glands is reduced to absent (3–5).

Iodide accumulation, the first step in the biosynthesis of the iodine-containing thyroid hormones, is mediated by the sodium/iodide symporter (NIS) (6). NIS is an integral basolateral plasma membrane glycoprotein that mediates—with remarkable affinity—sodium-coupled active iodide accumulation into the thyroid follicular cell (7). Structurally, NIS is a 13-transmembrane segment glycoprotein with an extracellular amino-terminus and a large intracellular carboxy-terminus, which contains a conserved monoleucine-based sorting motif that is required for NIS basolateral plasma membrane expression in the thyroid follicular cell (8, 9), and involved in reduced NIS plasma membrane expression in thyroid cancer (10). Recently, the three-dimensional structure of NIS was determined at atomic resolution using single-particle cryogenic electron microscopy, providing structural information on the mechanisms underlying NIS-mediated iodide transport (11), a long-standing question in the thyroid field.

Highlighting the importance of NIS in thyroid physiology, biallelic loss-of-function variants in the NIS-coding *SLC5A5* gene cause defective iodide accumulation, thus leading to congenital dyshormonogenic hypothyroidism (6). To date, more than forty pathogenic *SLC5A5* variants gene have been identified in patients with congenital dyshormonogenic hypothyroidism. Detailed molecular characterization of NIS variants has provided mechanistic information on structure-function relationships, highlighting critical amino acids for substrate binding, specificity, and stoichiometry, as well as folding and plasma membrane targeting (12–17).

Here, we conducted whole-exome sequencing in a patient with severe congenital dyshormonogenic hypothyroidism characterized by undetectable radioiodide accumulation in a eutopic thyroid gland and the salivary glands. A novel pair of compound heterozygous variants in the *SLC5A5* gene, c.1627G>A (p.G543R) and c.1684T>A (p.L562M), have been identified. The missense variant p.G543R in homozygous state has previously been associated with congenital hypothyroidism. Moreover, we conducted functional analysis revealing that the novel missense variant p.L562M lowers iodide accumulation due to defective NIS expression at the plasma membrane. In conclusion, we report novel compound heterozygous missense *SLC5A5* gene variants causing defective iodide accumulation, thus leading to congenital dyshormonogenic hypothyroidism.

## Materials and methods

### Patient’s medical records

The proposita was a full-term female infant born in 1993 as the third child of non-consanguineous and healthy Caucasian parents. The proposita was diagnosed with congenital hypothyroidism (TSH 60.0 µU/ml, range 1.3-10.0 µU/ml; total T_4_ 0.8 µg/dl, range 6.0-18.0 µg/dl) at 21 days of age, after detection of high TSH level on newborn screening (>60 µU/ml, cut off 15 µU/ml). Thyroid function analyses were performed by DELFIA system (PerkinElmer - Waltham, MA). Serum levels of thyroglobulin were not available. Clinical examination showed jaundice, lethargy, tongue protrusion, reticulated skin, and umbilical hernia. X-ray revealed lack of ossification of the distal femoral epiphysis. Levothyroxine replacement therapy was started immediately after diagnosis with a daily dose of 50 µg/kg with excellent treatment adherence over the course of 20-years follow-up period. Levothyroxine dosage was regularly adjusted based on thyroid function tests. At 2.3 years of age, thyroid function evaluation after levothyroxine withdrawal indicated permanent congenital hypothyroidism (TSH 99 µU/ml, range 0.5-6.5 µU/ml; total T4 1 µg/dl, range 4.5-12.5 µg/dl; free T4 0.1 ng/dl, range 0.8-2.2 ng/dl). Thyroid ultrasound revealed a eutopic small thyroid gland (0.23 ml, range 0.30-2.0 ml) with heterogeneous texture. Thyroid scintigraphy revealed undetectable ^131^I-iodide accumulation in the thyroid gland, as well as in the salivary glands, suggesting an iodide transport defect. Dosage of saliva-to-plasma radioiodide ratio was not technically available in the Division of Nuclear Medicine. At the age of 8, the Wechsler Intelligence Scale for Children (WISC-III) test showed normal neurocognitive development with lower performance in executive functions. The proposita grew normally in the 25^th^ percentile, underwent normal pubertal development, and reached a final height of 164 cm, according to the mean parental height. Parents and older sisters were clinically euthyroid.

### Ethical statement

The study was approved by the Ethics Committee of the Hospital de Niños Dr. Ricardo Gutierrez (Buenos Aires, Argentina) and was conducted with the written informed consent of the parents of the proposita’.

### Whole-exome sequencing

Genomic DNA was extracted from whole blood using Wizard Genomic DNA Purification Kit (Promega – Madison, WI). Whole-exome sequencing was performed by Macrogen (Seoul, South Korea) using an Illumina platform with 150-bp paired-end reads. Exome capture and library preparation were conducted using Agilent SureSelect V6 post (Agilent Technologies, Santa Clara, CA). Raw reads were mapped to the reference human genome (GRCh38) using the Burrows-Wheeler Alignment software and processed according to the recommendations of the Genome Analysis Toolkit. Variant calling was performed using HaplotypeCaller, and variant annotation was carry out using ANNOVAR. Precomputed scores from in silico meta-predictors able to reach strong evidence for pathogenicity (18), including BayesDel, REVEL, VEST4, and MutPred2, were annotated using dbNSFP (version 4.7a) database. Variant interpretation was determined according to the American College of Medical Genetics and Genomics guidelines in a quantitative Bayesian framework (19, 20).

### Sanger sequencing

The nucleotide sequence of coding exons of interest of the *SLC5A5* gene was determined by Sanger sequencing by capillary electrophoresis (Macrogen) as reported previously (21).

### Expression vectors and site-directed mutagenesis

The amino-terminus hemagglutinin (HA)-tagged human NIS cDNA sequence cloned into the pcDNA3.1 expression vector was kindly provided by Dr. Nancy Carrasco (Vanderbilt School of Medicine) (22).

Site-directed mutagenesis was conducted by PCR using the mutagenic oligonucleotides 5’-CCACTGTGCTGTGCAGAGCCCTCATCAGC (forward) and 5’-GCTGATGAGGGCTCTGCACAGCACAGTGG (reverse) for G543R, and 5’-CCCTGGCCCCGGGAATGTTGTGGTGG (forward) and 5’-CCACCACAACATTCCCGGGGCCAGGG (reverse) for L562M using Phusion Hot Start II DNA Polymerase (Thermo-Fisher Scientific - Waltham, MA), followed by methylated template plasmid digestion with DpnI (Promega – Madison, WI) (23). Mutagenic oligonucleotides were generated using QuickChange primer design software (Agilent Technologies - Santa Clara, CA). The fidelity of all constructs was verified by Sanger sequencing (Macrogen).

### Cell culture and transfections

HeLa cells (CCL-2, American Type Culture Collection, Rockville, MD) were cultured in Dulbecco Modified Eagle’s Medium (Thermo-Fisher Scientific) supplemented with 10% fetal bovine serum (Natocor, Córdoba, Argentina). Cells were transfected with 1 µg plasmid/well in 6-well plates using TurboFect Transfection Reagent (Thermo-Fisher Scientific). All experiments were conducted two days after transfection.

### ^125^I-iodide transport assays

Transfected cells were incubated in DMEM containing 10 μM iodide supplemented with 50 μCi/μmol ^125^I-iodide (PerkinElmer Life Sciences - Waltham, MA) for 30 min at 37°C (24). NIS-specific iodide uptake was assessed in the presence of 40 μM perchlorate. Intracellular radioiodide was extracted with ice-cold ethanol and quantified in a Triathler Gamma Counter (Hidex - Turku, Finland). The amount of DNA was determined by the diphenylamine method after trichloroacetic acid precipitation. Results were expressed as picomoles of iodide per μg DNA, and standardized by the ratio of the percentage of mutant NIS-positive cells to the percentage of WT NIS-positive cells—both of which were determined by flow cytometry under permeabilized conditions—in order to correct for differences in transfection efficiency between samples.

### Flow cytometry

Cells were fixed in 2% phosphate-buffered paraformaldehyde and stained with 0.5 μg/ml affinity-purified rabbit polyclonal anti-human NIS antibody (25) in PBS containing 0.2% human serum albumin and 0.2% Quillaja saponin (Sigma-Aldrich - St. Louis, MO) (26). After washing, cells were incubated with 1 μg/ml Alexa-488-conjugated anti-rabbit antibody (A-11008, Molecular Probes - Eugene, OR). The fluorescence of ~5x10^4^ events per tube was assayed in a BD FACSCalibur Flow Cytometer (BD Biosciences - San Jose, CA). Data analysis was performed with FlowJo software (Tree Star - Ashland, OR).

### Western blot

SDS-PAGE, electrotransference to nitrocellulose membranes, and immunoblotting were conducted as reported previously (26). Membranes were blocked and incubated with 0.2 μg/ml affinity-purified rabbit polyclonal anti-human NIS (25) and 0.2 μg/ml rabbit polyclonal anti-glyceraldehyde-3-phosphate dehydrogenase (GAPDH) (sc-25778, Santa Cruz Biotechnology - Santa Cruz, CA) primary antibodies. After washing, membranes were incubated with 0.07 μg/ml IRDye 680RD goat anti-rabbit (#926-68071) secondary antibody (LI-COR Biosciences - Lincoln, NE). Membranes were visualized by Odyssey Infrared Imaging System (LI-COR Biosciences). Relative band intensity was quantified using ImageJ software (National Institutes of Health - Bethesda, MD).

### Immunofluorescence

Transfected cells seeded onto glass coverslips were fixed in 2% phosphate-buffered paraformaldehyde and stained with 0.5 μg/ml affinity-purified rabbit polyclonal anti-human NIS antibody (25) and 2 μg/ml mouse monoclonal anti-Calnexin (sc-23954, Santa Cruz Biotechnology) antibodies in PBS containing 0.2% human serum albumin and 0.1% Triton X-100 for permeabilized conditions (27). Alternatively, cells were stained with 1:50 mouse monoclonal anti-human NIS VJ1 antibody (28) in PBS containing 0.2% human serum albumin for non-permeabilized conditions. Secondary staining was performed with 2 μg/ml anti-rabbit Alexa-488-conjugated and anti-mouse Alexa-594-conjugated antibodies (A-11008 and A-11012, Molecular Probes). Nuclear DNA was stained with 4′,6-diamidino-2-phenylindole (DAPI) (Molecular Probes). Coverslips were mounted with FluorSave Reagent (Calbiochem - La Jolla, CA) and images were acquired on an Olympus FluoView 1200 confocal microscope (Olympus America - Center Valley, PA). Quantification of NIS expression at the plasma membrane was conducted on non-permeabilized cells using FIJI ImageJ software (National Institutes of Health).

### Statistical analysis

Results are presented as the mean ± SEM of at least three independent experiments. Statistical tests were performed using Prism 8.0 software (GraphPad Software - La Jolla, CA). Multiple group analysis was conducted by one-way ANOVA and Newman-Keuls multiple-comparisons *post hoc* test. Differences were considered significant at *p*<0.05.

## Results

**Figure 1:**
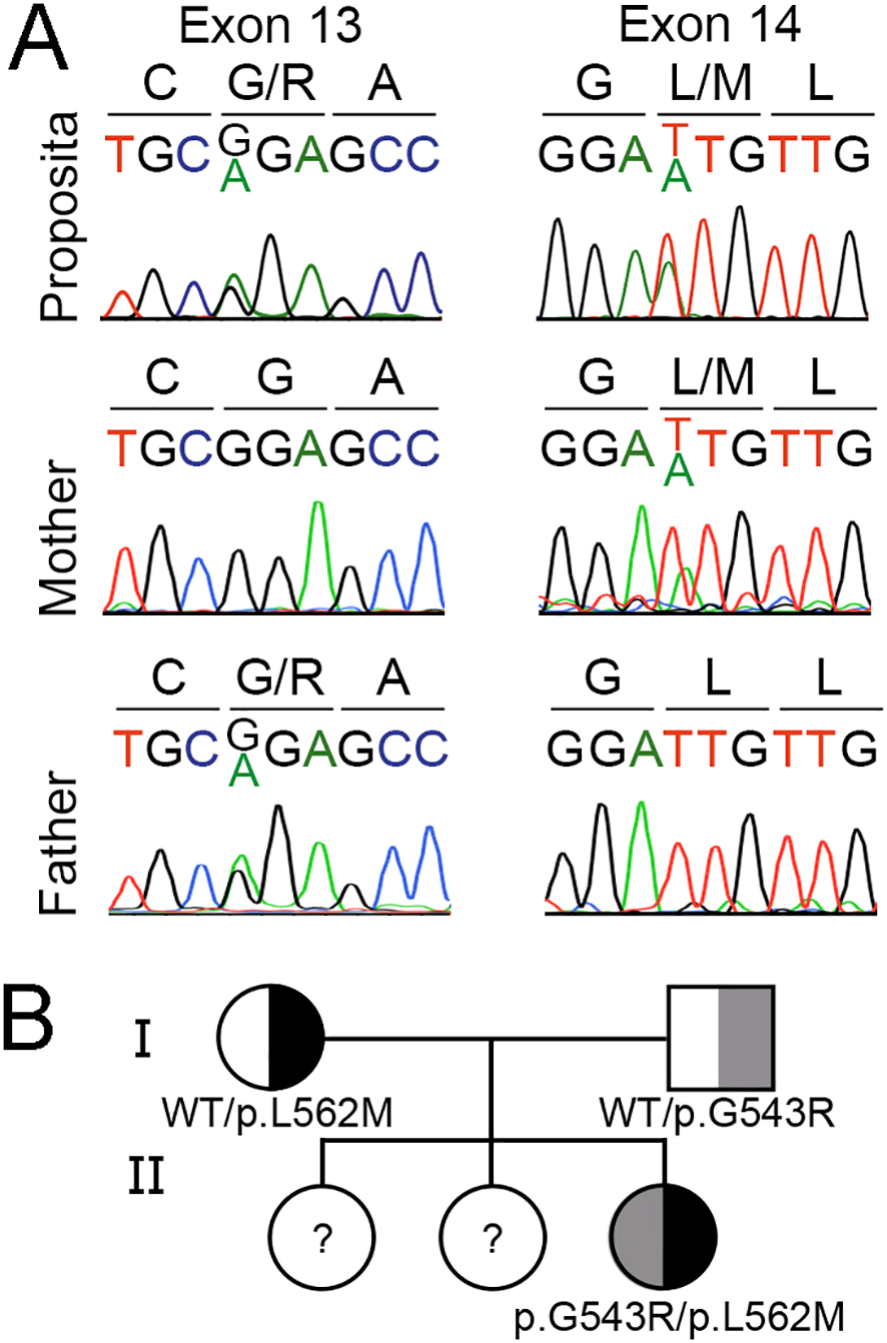
Identification of compound heterozygous missense *SLC5A5* gene variants causing congenital dyshormonogenic hypothyroidism. **(A)** Sanger sequencing chromatogram showing a 9-bp fragment of SLC5A5 exon 13 (nucleotides 1,624 to 1,632) and exon 14 (nucleotides 1681 to 1689). Amino acids are indicated using the one-letter code. The proposita is compound heterozygous for the variants c.1,627G>A (p.G543R) and c.1,684T>A (p.L562M), while the mother carries the variant c.1,684T>A and the father the variant c.1,627G>A. **(B)** Segregation of *SLC5A5* gene variants in the proposita’s family.

Iodide transport defect was suspected in the proposita on the basis of severe congenital dyshormonogenic hypothyroidism characterized by undetectable radioiodide accumulation in a eutopic thyroid gland, as well as in the salivary glands. Proposita´s whole-exome sequencing revealed heterozygous missense variants in the *SLC5A5* gene: c.1,627G>A, p.G543R and c.1,684T>A, p.L562M (GenBank Reference Sequence NM_000453.3, MANE Select Transcript). Pathogenic variants in other genes involved in thyroid development or physiology were not evidenced. The *SLC5A5* gene variants were further confirmed by Sanger sequencing (**Figure 1A**). Consistent with the recessive nature of the disease, analysis of the parents showed that the father is heterozygous for p.G543R and the mother for p.L562M, while older sisters were not included in the study (**Figures 1A, B**).

The variant c.1627G>A has been reported in Single Nucleotide Polymorphism database (rs776596656) in heterozygosis showing a total allele frequency of 0.00001054, according to The Genome Aggregation Database (version 4.1.0, acceded October 2024). In silico analysis predicted the p.G543R variant as pathogenic (**Table 1**). Significantly, the p.G543R variant was previously identified in homozygous state in a patient with goitrous congenital hypothyroidism (29), and functional characterization showed that G543R NIS lacked iodide transport activity due to impaired plasma membrane expression (30). The novel p.L562M variant was absent in The Genome Aggregation Database (version 4.1.0, acceded October 2024). In silico analysis using pathogenicity predictors revealed that the p.L562M variant did not meet the criteria to be considered either benign (BP4) or pathogenic (PP3) (**Table 1**) (18). According to the ACMG guidelines, the p.G543R variant was classified as pathogenic (PM2_Supporting, PM3_Supporting, PM5, PP3_Moderate, PP4, PS3), and the p.L562M variant as variant of unknown significance (PM2_supporting + PM3 + PP4).

**Table 1:** *In silico* pathogenicity prediction of variants.

Variant	BayesDel	REVEL	MutPred2	VEST4
p.G543R	0.28	0.78	0.85	0.95
p.L562M	-0.17	0.51	0.61	0.40

Pathogenicity (PP3)/benignity (BP4) evidence strength per variant was annotated using recommended thresholds for each meta-predictor (18). Thresholds for supporting levels of evidence for benignity (BP4) and pathogenicity (PP3) are ≤ -0.18 and ≥ 0.13 for BayesDel, ≤ 0.391 and ≥ 0.737 for MutPred2, ≤ 0.290 and ≥ 0.644 for REVEL, and ≤ 0.449 and ≥ 0.764 for VEST4.

**Figure 2:**
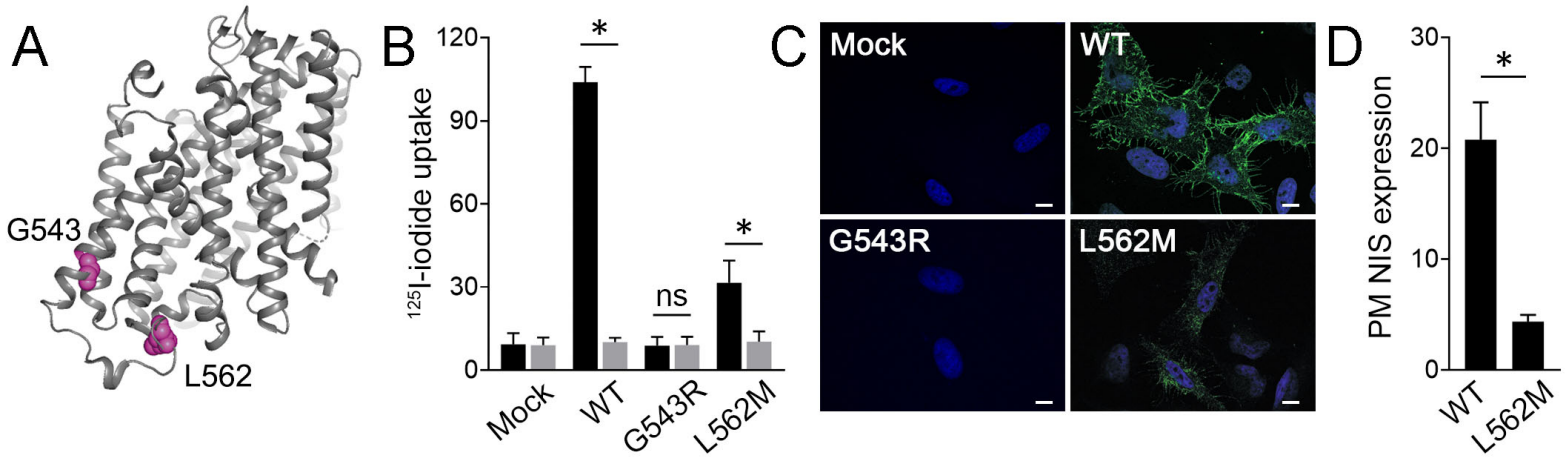
L652M severely reduces NIS plasma membrane expression. **(A)** Three-dimensional structure of rat NIS (PDB: 7UUY). The residues G543 and L562 are highlighted in pink. Image generated with The PyMOL Molecular Graphics System (Schrödinger, New York). **(B)** Steady-state iodide uptake in HeLa cells transiently expressing empty vector (mock) or WT, or the NIS variants G543R and L562M. Cells were incubated with iodide in the absence (black bars) or presence (gray bars) of perchlorate. Data are expressed in pmol of I^−^/μg of DNA ± SEM (n=3) and standardized by transfection efficiency, which was assessed by flow cytometry. *p<0.05 (ANOVA, Newman-Keuls test); ns, not significant. **(C)** Representative merged immunofluorescence analysis of WT or mutant NIS plasma membrane expression probed with anti-human NIS VJ1 antibody under non-permeabilized conditions. Cell nuclei were stained with DAPI (blue). Scale bars: 10 µm. **(D)** Mean fluorescence intensity quantification of immunofluorescence confocal microscopy images assessing plasma membrane NIS expression. Results are expressed as the mean ± SEM (n=10-12 cells). *p<0.05 (ANOVA, Student’s t-test).

According to the rat NIS three-dimensional structure (11), which shares 89% sequence identity with human NIS, the residue G543 is located on the cytoplasmic side of transmembrane segment XIII, while the residue L562 is located in the cytoplasm-facing carboxy-terminus (**Figure 2A**). Functional characterization was conducted to test the pathogenicity of the p.L562M variant. HeLa cells, which do not express NIS endogenously, transfected to express L562M NIS showed reduced perchlorate-sensitive iodide accumulation compared to cells expressing WT NIS (**Figure 2B**). Immunofluorescence confocal microscopy analysis under non-permeabilized conditions to assess plasma membrane NIS expression revealed that the levels of p.L562M NIS were significantly lower than those of WT NIS, suggesting that plasma membrane sorting of the mutant protein is severely impaired. (**Figures 2C, D**). Together, these findings indicate that the L562M substitution decreases NIS targeting to the plasma membrane, and consequently reduces NIS-mediated iodide transport. As previously reported (30), G543R NIS-expressing cells did not exhibit perchlorate-sensitive iodide accumulation, ultimately because G543R NIS was not targeted to the plasma membrane (**Figures 2B, C**).

**Figure 3:**
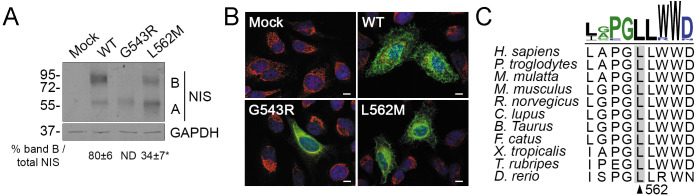
L562M reduces NIS exit from the endoclasmic reticulum. **(A)** Representative Western blot analysis of total lysates from transiently transfected HeLa cells probed with anti-human NIS and anti-glyceraldehyde-3-phosphate dehydrogenase (GAPDH) antibodies. Labels indicate the electrophoretic pattern of the corresponding NIS polypeptides (A: partially glycosylated; B: fully glycosylated) depending on glycosylation status. Quantification of fully glycosylated NIS polypeptides (band B) expressed as percent of total NIS is shown. Results are expressed as the mean ± SEM (n=3). *p<0.05 (ANOVA, Student’s t-test). **(B)** Representative merged immunofluorescence analysis of transiently transfected HeLa cells probed with anti-human NIS (green) and anti-human Calnexin (red) antibodies under permeabilized conditions. Cell nuclei were stained with DAPI (blue). Scale bars: 10 µm. **(C)** PSI/TM-Coffee-generated multiple amino acid sequence alignment of NIS orthologues from different metazoan species surrounding the amino acid L562 of human NIS. Sequence logo was generated using WebLogo 3 (https://weblogo.threeplusone.com).

On Western blots, the electrophoretic pattern of WT NIS showed a higher ratio of fully glycosylated polypeptides (~90 kDa, band B) to partially glycosylated polypeptides (~60 kDa, band A) (**Figure 3A**). Fully glycosylated NIS corresponds mostly to polypeptides located at the plasma membrane, whereas partially glycosylated polypeptides mostly correspond to those that have not exited the endoplasmic reticulum compartment. In contrast, the fully glycosylated NIS polypeptide (~90 kDa, band B) was minimally detected in L562M NIS-expressing cells, reinforcing the concept that only a small percentage of the mutant protein is targeted to the plasma membrane (**Figure 3A**). In addition, immunofluorescence confocal microscopy analysis under permeabilized conditions revealed that L562M NIS mostly colocalized with the endoplasmic reticulum-resident protein Calnexin, indicating that the mutant protein is mostly retained in the endoplasmic reticulum, whereas WT NIS was predominantly expressed at the plasma membrane (**Figure 3B**). As previously reported (30), G543R NIS is completely retained in the endoplasmic reticulum (**Figures 3A, B**). Multiple sequence alignment of NIS orthologues from different metazoan species revealed that the p.L562M variant affects a highly conserved residue (**Figure 3C**). Together, these findings suggest that the p.L562M variant decreases NIS targeting to the plasma membrane, and consequently reduces NIS-mediated iodide transport, thereby expanding the spectrum of congenital hypothyroidism-causing *SLC5A5* gene variants.

## Discussion

Next-generation sequencing has been instrumental in expanding the mutational landscape of monogenic forms of congenital hypothyroidism. In particular, whole-exome sequencing analysis has revealed pathogenic variants in novel genes involved in the pathogenesis of congenital hypothyroidism (31, 32). Here, using whole-exome sequencing, we identify a novel pair of compound heterozygous missense *SLC5A5* gene variants—p.G543R and p.L562M—in a proposita with congenital dyshormonogenic hypothyroidism characterized by undetectable radioiodide accumulation in a eutopic thyroid gland, as well as in the salivary glands, suggestive of an iodide transport defect phenotype.

The p.G543R NIS variant was previously identified in homozygosity in a patient with goitrous congenital hypothyroidism (29). In addition, another variant in the same residue, p.G543E, was detected in homozygosity in two siblings with goitrous congenital hypothyroidism who showed minimal radioiodide accumulation in the thyroid gland (33). Functional characterization of the G543R/E variants revealed that they impair NIS exit from the endoplasmic reticulum, apparently due to a folding defect, thus reducing NIS-mediated iodide accumulation (30). The novel variant reported here, p.L562M, severely reduced normal iodide accumulation by repressing NIS transport to the plasma membrane. Of note, the functional evaluation supported the classification of the p.L562M NIS variant as likely pathogenic (PS3 criteria added), thus our data highlight the importance of interpreting congenital hypothyroidism-associated variants with caution according to consensus guidelines.

The residue L562 is located in the intracellularly-facing carboxy-terminus, which is required for the expression of NIS at the basolateral plasma membrane in the thyroid follicular cell (8). Underscoring the significance of the carboxy-terminal region, the missense p.S547R and p.G561E variants and the nonsense p.R636*, which generates a truncated protein missing the last eight amino acids, identified in patients with congenital dyshormonogenic hypothyroidism causes the intracellular retention of the mutant protein (34–36). Of note, the molecular characterization of the iodide transport defect-causing p.G561E NIS variant, which is adjacent to the p.L562M variant reported here, revealed the importance of a highly conserved [L/M]xW[D/E] tryptophan-acidic sorting motif involved in NIS transport to the plasma membrane (36). The p.G561E variant shifts the equilibrium of the adjacent unstructured tryptophan-acidic motif towards a structured alpha-helical conformation reducing its recognition by the kinesin-1 subunit kinesin light chain 2, thereby interfering with NIS maturation beyond the endoplasmic reticulum, and reducing iodide accumulation (36).

Considering that NIS-mediated iodide accumulation is the first step in the synthesis of iodine-containing thyroid hormones, our results suggest that the pair of compound heterozygous missense NIS variants p.G543R and p.L562M impairs normal iodide accumulation by interfering with NIS maturation and transport to the plasma membrane. Notably, the *in vitro* data correlate with clinical findings of impaired radioiodide accumulation in the thyroid when the patient was evaluated by thyroid scintigraphy. Therefore, the consequent lack of sufficient NIS molecules at the basolateral plasma membrane of thyroid follicular cells reveals the mechanism underlying the deficient iodide accumulation leading to congenital dyshormonogenic hypothyroidism.

## Data Availability

The raw data from whole exome sequencing remain confidential due to ethical considerations. All additional raw data supporting the conclusions of this article will be made available by the authors, without undue reservation.
